# An Eye Movement Monitoring Tool: Towards a Non-Invasive Device for Amblyopia Treatment

**DOI:** 10.3390/s25154823

**Published:** 2025-08-06

**Authors:** Juan Camilo Castro-Rizo, Juan Pablo Moreno-Garzón, Carlos Arturo Narváez Delgado, Nicolas Valencia-Jimenéz, Javier Ferney Castillo García, Alvaro Alexander Ocampo-Gonzalez

**Affiliations:** 1 Faculty of Engineering, Universidad Santiago de Cali, Cali 760035, Colombia; juan.castro17@usc.edu.co (J.C.C.-R.); juan.moreno15@usc.edu.co (J.P.M.-G.);; 2 Telecommunications Laboratory (LABTEL), Electrical Engineering Department, Federal University of Espírito Santo (UFES), Vitória 29075-910, ES, Brazil; 3 Department of Education of Santiago de Cali, Alcaldía de Santiago de Cali, Cali 760035, Colombia; d.anc.nicolas.valencia@cali.edu.co; 4 Faculty of Engineering and Basic Sciences, Universidad Autónoma de Occidente, Cali 760035, Colombia; jfcastillo@uao.edu.co; 5 Faculty of Health, Universidad Santiago de Cali, Cali 760035, Colombia

**Keywords:** amblyopia, visual therapy, non-invasive, eye tracking, monitoring tool, dichoptic therapy

## Abstract

Amblyopia, commonly affecting children aged 0–6 years, results from disrupted visual processing during early development and often leads to reduced visual acuity in one eye. This study presents the development and preliminary usability assessment of a non-invasive ocular monitoring device designed to support oculomotor engagement and therapy adherence in amblyopia management. The system incorporates an interactive maze-navigation task controlled via gaze direction, implemented during monocular and binocular sessions. The device tracks lateral and anteroposterior eye movements and generates visual reports, including displacement metrics and elliptical movement graphs. Usability testing was conducted with a non-probabilistic adult sample (*n* = 15), including individuals with and without amblyopia. The System Usability Scale (SUS) yielded an average score of 75, indicating good usability. Preliminary tests with two adults diagnosed with amblyopia suggested increased eye displacement during monocular sessions, potentially reflecting enhanced engagement rather than direct therapeutic improvement. This feasibility study demonstrates the device’s potential as a supportive, gaze-controlled platform for visual engagement monitoring in amblyopia rehabilitation. Future clinical studies involving pediatric populations and integration of visual stimuli modulation are recommended to evaluate therapeutic efficacy and adaptability for early intervention.

## 1. Introduction

Early and periodic visual examinations in children help ensure the early diagnosis of any stimulus deprivation, refractive error, or strabismus, all of which are factors causing amblyopia [1,2]. The global prevalence of amblyopia is estimated to be around 1–5% [3]. According to the World Health Organization (WHO), approximately 19 million children under the age of 15 suffer from visual impairment, with 12 million of them experiencing uncorrected refractive errors and amblyopia [4]. Occasionally, amblyopia does not present in an obvious manner, which requires the need for visual anticipative assessment to determine if the infant at that early age is or will be close to suffering it [5,6].

Due to the presence of amblyopia in early life, sometimes known as “lazy eye”, visual problems such the loss of cortical visual development in one or both eyes can be caused [7,8,9]. Amblyopia typically appears during childhood when the developing visual system fails to transmit a clear image to the visual cortex [10,11]. It is diagnosed by determining a decrease in visual acuity in one or both eyes that is not in proportion to the structural abnormality of the eye and by ruling out any other visual disorder as an underlying cause [12].

Optimal visual rehabilitation for children diagnosed with amblyopia typically aims for progressive improvement in ocular function over time; however, this objective is often unmet [13,14]. One significant barrier to achieving successful outcomes lies in the prohibitively high cost of medical equipment required for active vision therapies, rendering them inaccessible to a majority of individuals [15]. Children with newly diagnosed amblyopia are more likely to come from families with low socioeconomic status [15]. Consequently, rehabilitation efforts often resort to visual exercises administered by parents or caregivers, albeit with diminished efficacy and limited capacity for comprehensive monitoring of the underlying pathology [16].

In addition, the viability of alternative rehabilitation modalities, such as occlusion therapies employing patches or corrective lenses to encourage visual engagement of the amblyopic eye, are constrained by challenges related to low compliance and resistance among certain infants [17,18]. These interventions often elicit discomfort and irritation, leading to diminished acceptance by young patients [19]. Furthermore, prolonged reliance on occlusion therapy carries the risk of inducing reverse amblyopia, where the previously healthy eye experiences a decline in visual function [14,20]. In response to these limitations, dichoptic digital therapy approaches have focused on promoting balanced binocular visual interaction by presenting separate visual stimuli to each eye [16]. By adjusting parameters such as image contrast or using dissociative lenses, these methods aim to reduce interocular suppression and foster more natural visual development, offering a less invasive and more engaging alternative to traditional patching [21].

Moreover, the creation of a device designed to mitigate the risk of inducing reverse ocular conditions through prolonged therapeutic interventions, such as occlusion therapy, would likely foster greater participation in rehabilitation among children and adolescents, alleviating apprehensions associated with potential adverse effects. As highlighted by the World Health Organization (WHO), a staggering 45% of visual impairment cases could have been averted through timely access to affordable visual rehabilitation therapies [4].

Dichoptic therapy, perceptual learning, anaglyphic glasses, certain video games, and specialized serious games, including virtual reality systems tailored for amblyopia treatment, have demonstrated efficacy in addressing this condition during therapy sessions [16,21,22,23]. Consequently, the pursuit of strategies facilitating non-invasive visual rehabilitation for children remains imperative [16].

Therefore, there is a pressing need for the development of supportive devices that can assist in pediatric amblyopia management by enhancing patient engagement and adherence to therapy, while minimizing the risk of adverse ocular effects associated with traditional treatments. Such devices should prioritize child comfort, intuitive interaction, and non-invasive monitoring strategies to facilitate their widespread acceptance and use [24,25,26]. In this context, the proposed Device for Recording and Analysis in Amblyopia Management (DRAM) aims to leverage eye-tracking technology to monitor ocular movements during interactive visual tasks without requiring occlusion of either eye. Through the use of pre-designed on-screen mazes inspired by contemporary dichoptic training principles, the system enables engagement assessment and progress monitoring during rehabilitation sessions, as will be described in the following sections. By addressing these considerations, DRAM is positioned as a complementary tool that could support early interventions in visual health and enhance adherence to amblyopia therapies, particularly in settings where access to specialized care is limited.

Building on the identified needs and challenges in pediatric amblyopia treatment, this study focuses on the development of a pupil detection classifier and its integration into a low-cost, user-friendly device designed for supportive eye monitoring and training. The current work prioritizes technical validation and usability evaluation, establishing foundational evidence for the device’s functionality. Initial testing was conducted with a non-probabilistic sample population of adults, setting the stage for future clinical trials aimed at assessing efficacy, safety, and adaptability in pediatric populations.

## 2. Challenges and Constraints of Healthcare Tools and Therapies for Amblyopia

Despite the challenges, significant progress has been made in the field, as evidenced by various studies and the development of innovative treatment methods. Research into therapies for amblyopia spans both children and adults. Table 1 emphasizes the diversity of approaches, including occlusive therapy, dichoptic therapy, and emerging virtual reality-based interventions. By presenting key findings, such as the significant improvements in visual acuity and stereoacuity achieved through these therapies, the table underscores the advancements and challenges in amblyopia treatment. Its inclusion highlights the necessity for innovative and patient-centered therapeutic devices, aligning with the rationale for the proposed system.

In addition to therapeutic advancements, studies have also explored methods to assess the characteristics of amblyopia. The Hirschberg reflex test is a widely used diagnostic tool for measuring visual acuity and evaluating ocular alignment, aiding in the identification of conditions such as esotropia (inward eye deviation) [34]. Similarly, the TNO test, developed by The Netherlands Organization, is a prominent method for detecting binocular vision defects (stereopsis) in young children aged from two and a half to five years [35]. In [36], various neural-level hypotheses have been reviewed to explain amblyopia, including interocular competition and cortical suppression, alterations in synaptic plasticity, imbalance in cortical neuronal activity, disruption in the organization of ocular dominance columns, deficits in high-level visual integration, and alterations in functional connectivity. Specifically, the use of magnetic resonance imaging (MRI) in studies of monocular amblyopia has suggested a reduction in cortical volume in the occipital regions of the left hemisphere, as well as a cortical decrease in both hemispheres at the level of the inferior temporal gyrus. Furthermore, a study employing surface-based morphometry to analyze the cortex of individuals with anisometropic amblyopia revealed a reduction in the primary visual cortex (Brodmann area 17), suggesting that a thinner primary visual cortex is associated with more severe symptoms of amblyopia [37]. These diagnostic approaches complement the therapeutic methods summarized in Table 1, providing a foundation for tailored and effective interventions.

Beyond diagnostic tests, there are also treatments involving eye movement, which may include occupational therapies. For example, a scheme involving a video eye tracker that combines an artificial eye for eye movement testing in children aims to improve diagnostic criteria for amblyopia and provide theoretical support by using eye movement data for the development of deep learning diagnostic and predictive applications [38]. The validation of this study enrolled 198 children of whom 92 were in the amblyopia group and had a mean age of 5.93 years. High-resolution video-based eye trackers such as Eyelink 1000 have also been used to measure vertical and horizontal eye movements in order to examine the relationship of PEFs to amblyopia optotype acuity and ocular contrast sensitivity deficits [39]. This study involved 25 amblyopic subjects of whom six had anisometropia and ten had strabismus. Eye tracking responses (OFRs), which are short-latency reflexive eye movements, have been found to be sensitive to changes in interocular correlation, making them useful for the assessment of stereoscopic deficits. Therefore, these OFRs could improve the results of treatments for amblyopia and strabismus. In this study, OFR measurements due to dichoptic stimulations are performed in children with compromised stereopsis due to the amblyopia condition and with normal stereopsis, using customized high-resolution video eye tracking equipment [40] where two groups of six children participated, with an age between 7 and 12 years old. The results suggest that there is potential for improvement of early diagnosis and treatment outcomes of amblyopia.

Eye movement appears to be associated with visual acuity. Several studies investigating visual acuity have found a correlation between amblyopia and eye movement. Individuals with low vision due to macular diseases or amblyopia may exhibit abnormalities in their fixational eye movements (FEMs), leading to unstable ocular fixation, which in turn reduces visual acuity. Therefore, eye movements can provide insight into the extent of visual acuity or stereopsis loss in an individual [41,42]. Other studies indicate that visual acuity is a key limiting factor for ocular fixation in adults. A significant correlation has been demonstrated between visual acuity and eye movements in individuals with amblyopia, suggesting that improving fixation stability should be a primary goal in amblyopia treatment. For instance, visual disorders such as strabismus impair motor coordination of eye movements, leading to fixation instability and disconjugate ocular movements [43,44]. During fixation, involuntary eye movements such as microsaccades, saccades, drift, and tremor persist even when one attempts to maintain steady gaze [45]. FEMs in both children and adults shift retinal images between the two eyes to achieve fixation disparity, defined as the positional difference between the eyes. Normal binocular vision is maintained through coordinated ocular motor control, fine-tuning mechanisms, and sensory integration [46]. Furthermore, it has been found that the visual acuity of the amblyopic eye affects the composite amplitude of rapid eye movements. One study reported that amblyopic eye acuity significantly influenced the variance in eye position [47].

## 3. DRAM System Overview

This section provides an in-depth description of the materials, methodologies, and design considerations involved in the development of the device, addressing the challenges previously identified and focusing on its application in ocular therapies. Specifically, the integration of computer vision and distance calculation techniques is explained, demonstrating how the distance sensor and OLED display are utilized to support therapeutic interventions. The functional operation of the system is further illustrated through a flowchart. Additionally, the data collection, storage, and representation processes are detailed, ensuring a comprehensive understanding of how the device monitors and evaluates therapeutic progress. The section concludes with an overview of the participant selection process for the operational test and the subsequent usability survey designed to collect qualitative feedback.

The device design was guided by the specific needs of children with visual impairments, addressing the discomfort and potential adverse effects associated with traditional therapies. Families often express concerns about the prolonged use of such therapies, as they may lead to complications like reverse amblyopia [48]. Furthermore, dichoptic therapies, despite their effectiveness, remain financially inaccessible for many, leaving families to rely on traditional methods that may pose risks and limitations.

To address these challenges, the device incorporates an active therapy approach based on the resolution of a maze through eye movements [49]. Therapy begins with monocular sessions, where the healthy eye is covered and the user completes the maze by performing eye movements with the amblyopic eye. This approach is supported by evidence suggesting that the combination of occlusion with active therapy improves visual function in various types of amblyopia, offering a holistic solution for visual rehabilitation [50]. During these sessions, metrics such as maze completion time and number of wall collisions are recorded to stimulate visual engagement in the affected eye and prevent cortical suppression.

Once the specialist has determined sufficient improvement in the amblyopic eye, therapy moves to binocular sessions. This phase uses the eye movement of both eyes to complete the maze, promoting the synchronization of visual information between the eyes and the brain. Binocular therapy has shown promising results in increasing eye coordination and neuronal plasticity through attractive visual environments or games [51]. All therapeutic sessions are held at a distance of 40–50 cm from the maze to ensure optimal conditions for therapy. The therapy aimed to improve adherence due to the integration of the maze on the screen, in which eye movement is recorded to solve the maze.

### 3.1. Hardware Design

The hardware of the device is centered around the ESP32-CAM microcontroller, chosen for its versatility and ability to function as an IP camera within a private network. This feature enables the real-time capture of the user’s facial images, which is crucial for monitoring and adapting the therapy process. Simultaneously, a 128 × 64 OLED display is integrated to present a visual maze that serves as the core of the therapeutic activity.

To ensure adherence to the recommended therapeutic distances specified by specialists, the device incorporates a VL53L0X laser distance sensor. This sensor is known for its exceptional precision, capable of measuring distances ranging from 4 mm to 2000 mm. It employs a Class 1 laser, certified under IEC 60825-1:2014—Third Edition, guaranteeing ocular safety during operation [52]. The laser sensor plays a critical role in maintaining the proper distance between the user and the device, ensuring both therapeutic effectiveness and user safety.

Communication between the microcontroller, the laser distance sensor, and the OLED display is facilitated by the I2C communication protocol, a robust and efficient interface for connecting multiple components. Power is supplied through a 3800 mAh power bank, regulated to 5 V, which allows the device to function independently of external power sources. This portable power solution enhances usability, providing flexibility for various therapy settings.

All hardware components are securely housed in a 3D-printed case, designed to ensure durability, ergonomics, and ease of handling. The compact and user-friendly design supports seamless integration into therapeutic sessions. A conceptual diagram of the hardware configuration will be presented later in the document, illustrating how these components interact to deliver a cohesive and effective ocular monitoring experience.

### 3.2. Software Design

The device’s software (version 2.1) integrates advanced image processing and real-time communication features to facilitate ocular therapy sessions. The ESP32-CAM microcontroller (Ai-Thinker, Shenzhen, China) captures images of the user, which are transmitted via Wi-Fi to a custom-designed application. This application performs real-time pupil tracking, estimating the user’s gaze direction based on lateral and anteroposterior displacements of the pupil. Anteroposterior displacement is a movement from the front to the back of an object or body.

**Figure 1:**
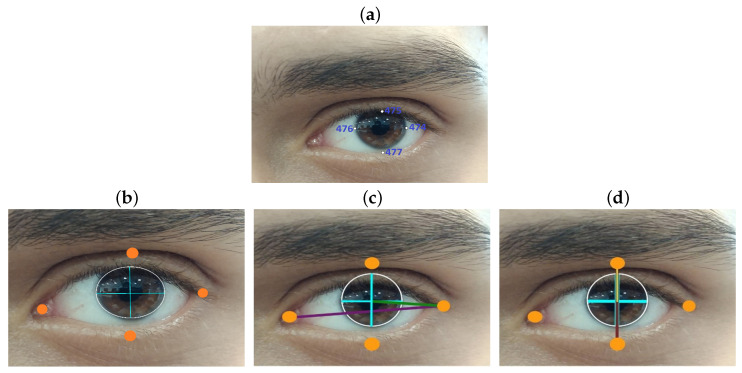
(**a**) Facial points for left iris recognition. (**b**) Key points location. (**c**) Calculation of the distance horizontally traversed by the pupil. (**d**) Calculation of the distance traveled vertically by the pupil.

Pupil detection is achieved using eight reference points derived from the facial landmarks provided by the MediaPipe library, outlining the iris of both eyes. Specifically, points 469, 470, 471, and 472 are used for the right eye, while points 474, 475, 476, and 477 are employed for the left eye (Figure 1a). The contour formed by these points is analyzed using the OpenCV function “minEnclosingCircle,” which calculates the circle encompassing the iris. This provides the precise “x” and “y” coordinates of the pupils, located at the circle’s center.

To determine gaze direction, four additional landmarks are selected for each eye to outline the eye contour. The maximum horizontal distance between the lateral eye landmarks is calculated, as well as the horizontal distance between the pupil and the external lateral landmark of the eye contour. These distances are represented in Figure 1c, with the maximum horizontal distance shown as a purple line and the distance between the pupil and the external landmark as a green line.

Similarly, anteroposterior displacement is calculated by determining the maximum vertical distance between the upper and lower landmarks of the eye contour. The displacement is then derived by subtracting the distance of the pupil from the upper landmark, as depicted in Figure 1d, where the vertical reference distances are shown as a brown line, and the pupil-to-landmark distance is represented by a yellow line.

**Figure 2:**
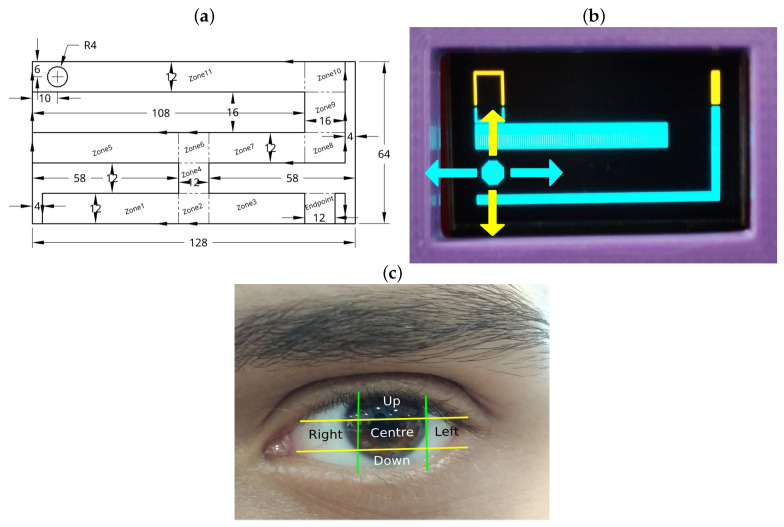
(**a**) Level 5 layout with zone distribution. (**b**) Directions on screen. (**c**) Eye zones.

The software incorporates a progressive therapy structure comprising five levels. Each level requires the user to complete tasks by navigating a maze through gaze-controlled movements, simulating a joystick system. The four basic movements (up, down, left, and right) are achieved by the user’s gaze direction, allowing the user to guide a ball through the maze. The user’s progress is tracked by recording the time taken to pass through three key zones in each level. For instance, in level 1, the key zones include zones 2, 4, and the finish line; in levels 2 and 4, zones 3, 7, and the finish line; in level 3, zones 6, 11, and the finish line; and in level 5, zones 9, 6, and the finish line. The design and distribution of these zones are illustrated in Figure 2a.

The gaze-controlled navigation is defined by dividing the user’s eye region into five zones. Depending on the pupil’s location, the device executes one of four actions: upward movement (“Up” zone), downward movement (“Down” zone), rightward movement (“Right” zone), or leftward movement (“Left” zone). If the pupil is detected within the center zone, the ball remains stationary. This division enables precise control, enhancing the user’s ability to navigate the maze while exercising the amblyopic eye. Figure 2b demonstrates the corresponding ball movements, while Figure 2c illustrates the segmentation of the eye region.

To synchronize commands between the graphical interface and the device, WebSocket communication is implemented. This protocol ensures seamless data transfer, allowing the device to receive gaze-based commands and send crucial therapy data, such as distance measurements from the VL53L0X sensor, the user’s current level, the number of collisions or errors, and the real-time position of the ball within the maze. This efficient and reliable communication system underpins the interactive functionality of the device, optimizing its therapeutic effectiveness.

### 3.3. DRAM Usage

**Figure 3:**
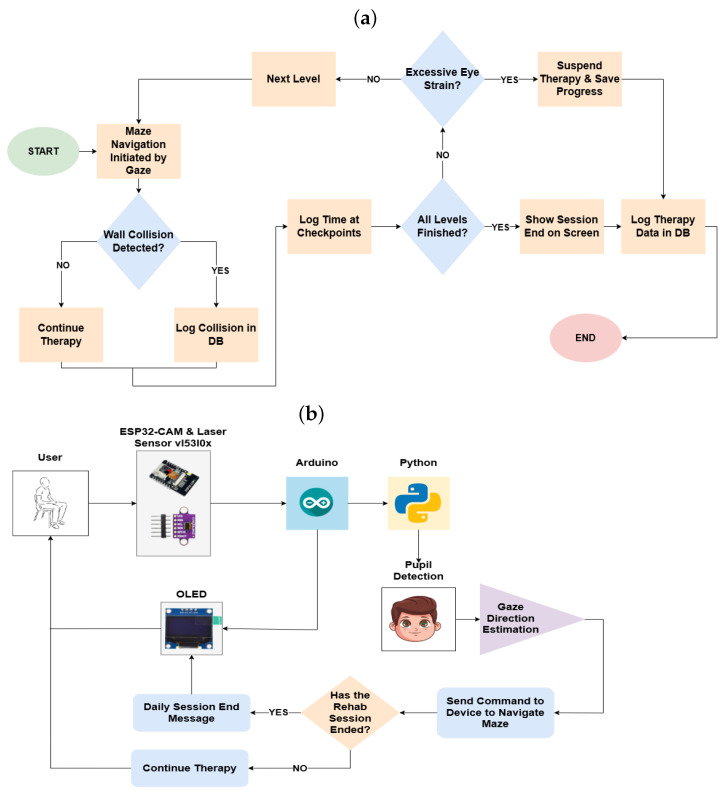
(**a**) Operating flowchart. (**b**) Conceptual diagram.

The operation of the device follows a systematic process outlined in Figure 3a, which depicts the workflow via a flowchart.

To begin, the device is powered on and connected to a Wi-Fi network. The user accesses the graphical interface through a compatible device, logging in with their unique credentials. If the user is not yet registered, they are required to create a new profile, enabling individualized session tracking. Once logged in, the user selects the type of therapy to be conducted—monocular or binocular—and positions themselves at a distance of 40 to 50 cm from the device.

At this stage, the first level of the therapy maze is displayed on the OLED screen of the device. Simultaneously, the ESP32-CAM module begins capturing real-time images of the user’s face while gathering distance measurements through the VL53L0X sensor. These data, along with information such as the current level, user position, and instances of collisions with maze walls, are transmitted via WebSocket communication to the graphical interface.

Upon verifying the user’s correct positioning, the therapy session can be initiated by clicking the “Start Therapy” button on the interface. Figure 3b provides a conceptual diagram illustrating the operational flow.

**Figure 4:**
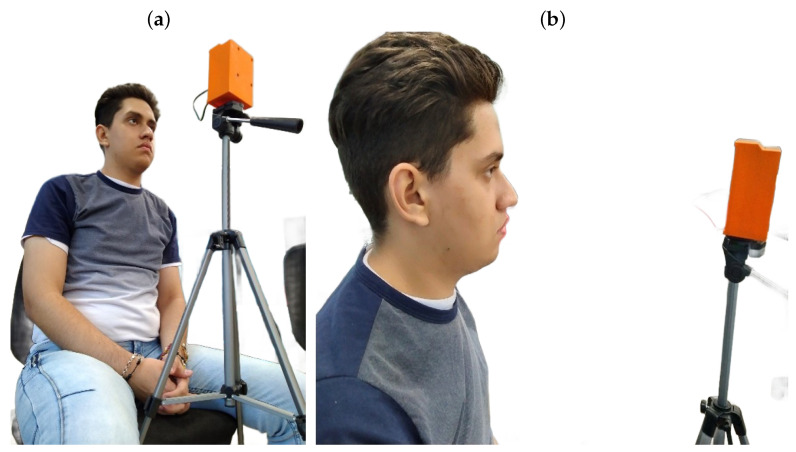
(**a**) Location in front of the visual device. (**b**) Distance in front of the visual device.

To ensure optimal functionality of the device during each session, the following steps should be adhered to:

1. Proper Positioning: The user should be seated in a comfortable and upright position, maintaining a distance of 40 to 50 cm from the device. The device height must be adjusted to enable precise pupil tracking, as illustrated in Figure 4.
2. Maze Completion: The user is required to navigate through the proposed mazes by controlling the on-screen circle with lateral and anteroposterior eye movements. To achieve accurate tracking, the user’s head must remain stationary, with only the eyes in motion.
3. Session Duration: The user should maintain their position until they have completed all the maze levels or feel fatigued. If unable to complete the session, the progress made up to the last level will be recorded in the database.
4. Progression and Duration: Therapy begins with a simple maze and gradually increases in complexity throughout the session. The total duration of the therapy is estimated to range between 6 and 8 minutes, encompassing all five levels programmed into the device. The frequency of therapy sessions is determined by a specialist based on the severity of the amblyopia.

Figure 4a depicts the correct user positioning in front of the device, while Figure 4b illustrates the optimal distance for accurate visual therapy. It is also noted that the individual shown in the figure approved their inclusion in the publication.

By following these guidelines, the device allows for a systematic and individualized approach to vision therapy, ensuring accurate data collection and effective therapy sessions tailored to the specific needs of the user.

### 3.4. Report Generation

The monitoring device provides detailed results on the lateral and anteroposterior displacements of the user’s eyes. These measurements allow the evaluation of improvements in eye movement and navigation time across various designed mazes for therapy sessions. Progress observed through these metrics can indicate enhanced visual acuity in the user, complementing specialist evaluations.

#### 3.4.1. Lateral and Anteroposterior Displacement

Eye movements are represented through ellipse graphs, illustrating the displacements during each session. These graphs highlight the maximum and minimum lateral and anteroposterior movements, recorded separately for each eye in “Monocular” therapy and as an average for both eyes in “Binocular” therapy. By comparing ellipses from multiple sessions, changes in the user’s eye movements can be tracked over time.

The graphing process relies on parameters outlined in the theoretical framework, where variations in these parameters modify the ellipse shape. For example, *X_min_* and *X_max_* denote the minimum and maximum lateral displacements, while *Y_min_* and *Y_max_* represent the corresponding anteroposterior displacements. These variations allow a thorough analysis of changes in the amblyopic eye’s movement.

Additionally, a graphical representation of the eye movements over time is generated, providing insight into the extent of lateral and anteroposterior displacements during specific intervals of the session.

#### 3.4.2. Linear Regression

The device tracks errors made by the user when colliding with maze walls, categorized by level and session total. This enables an analysis of whether the user reduces errors as they progress through therapy. Key maze areas, referred to as “checkpoints” record the time when the user reaches these points, along with the total time taken to complete the maze.

**Figure 5:**
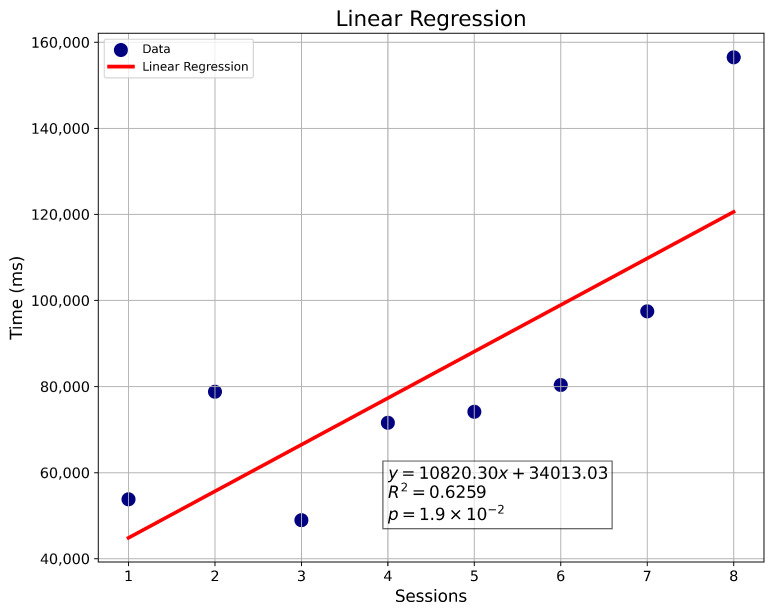
Linear regression of sessions vs. time (ms).

Using these data, weekly linear regressions are generated to assess user progress. Metrics include the number of errors per level and the time required to complete each maze, allowing trends in user performance to be visualized. Regression graphs also include values such as the coefficient of determination (R2), which is the proportion or percentage of variation in the dependent variable [53]. The *p*-value is also shown, which is a probabilistic measure linked to evidence against the null hypothesis, where traditionally a *p*-value lower than 0.05 is considered a threshold for exposing statistical significance [54]. An example of such analysis is shown in Figure 5.

#### 3.4.3. Data Tables

All data related to lateral and anteroposterior eye movements, maze completion times (in milliseconds), and errors are stored in a relational database. This database organizes and relates data points, associating them with individual users. Users can access their recorded data for each therapy session, enabling them to monitor their progress over time.

### 3.5. Population

A non-probabilistic sampling method was employed to select the population for this study, primarily due to the challenges associated with testing electronic devices on pediatric populations. Additionally, it is essential to recognize that significant improvements in amblyopia often require prolonged ocular monitoring guided by specialized professionals. Consequently, this study focuses on evaluating the functionality of the device rather than its long-term therapeutic impact.

Despite these constraints, functionality tests were conducted with a population that included individuals diagnosed with amblyopia. Specifically, the tests involved 15 young adults with the following age distribution: one participant aged 18, two aged 19, two aged 21, five aged 22, three aged 24, one aged 25, and one aged 27.

The tested population consisted of diverse visual conditions:

- Two participants with myopia.
- One participant with astigmatism.
- Three participants with amblyopia:
  User ID 5 presented with strabismic amblyopia in the left eye.
  User IDs 15 and 8 exhibited amblyopia in the right eye.
- The remaining participants had no diagnosed ocular conditions.

By involving a varied group of users, including individuals with and without ocular conditions, the study was able to assess the device’s functionality under diverse scenarios. These results demonstrate the device’s potential applicability to real-world therapy contexts, offering an innovative and accessible tool to complement traditional treatments for amblyopia.

### 3.6. Ethical and Safety Considerations

Prior to conducting tests with the population described in the previous section, all participants were required to complete an informed consent form. This document outlined key aspects such as potential risks or discomforts, the objectives and justification of the project, the procedures to be undertaken, and other relevant details. The process adhered to the ethical principles established by the UNESCO Budapest Declaration, underscoring the critical importance of integrity and ethical conduct in scientific research.

The informed consent form served as a vital mechanism to ensure that participants voluntarily and knowingly agreed to undergo the specified procedures. Participants were informed of potential risks, including mild dizziness or eye discomfort caused by repetitive movements during therapy. They were assured that if they experienced any adverse symptoms and were unable to continue, they could freely terminate the session. In such cases, the data collected up to that point would still be retained for analysis.

To safeguard participant anonymity, a unique identification (ID) was assigned to each individual, ensuring their personal information remained confidential when presenting results.

This study was rigorously reviewed and approved by the Research Ethics Committee of the Faculty of Engineering at Universidad Santiago de Cali (USC). Approval was granted under Minute No. 87 of Session 042, held on 20 June 2023.

By adhering to these ethical and safety standards, the study ensures the rights, dignity, and well-being of all participants while maintaining the scientific validity of its findings.

## 4. Results

In addressing the identified problem, a visual monitoring device was successfully designed, as detailed in the previous section. This section outlines the outcomes of its implementation, categorized into three key areas: usability testing, data visualization through graphs and tables generated by the graphical interface, and a description of the device’s technological characteristics. Figure 3b provides a conceptual diagram summarizing the technologies involved in developing the device.

To evaluate the effectiveness and usability of the monitoring device, a non-probabilistic convenience sampling method was employed, involving 15 participants. The primary selection criterion was the presence of amblyopia or related visual disorders. However, individuals without these conditions but with the willingness and availability to participate were also included. A video demonstrating the device’s operation is available via the following link: https://goo.su/DRir (accesed in September 2023).

### 4.1. Eye Movement and Maze Completion Time Report

Session reports for each user were generated through the graphical interface used for the therapy. Users were required to log in with their registered credentials to access therapy modules and review their session outcomes.

**Figure 6:**
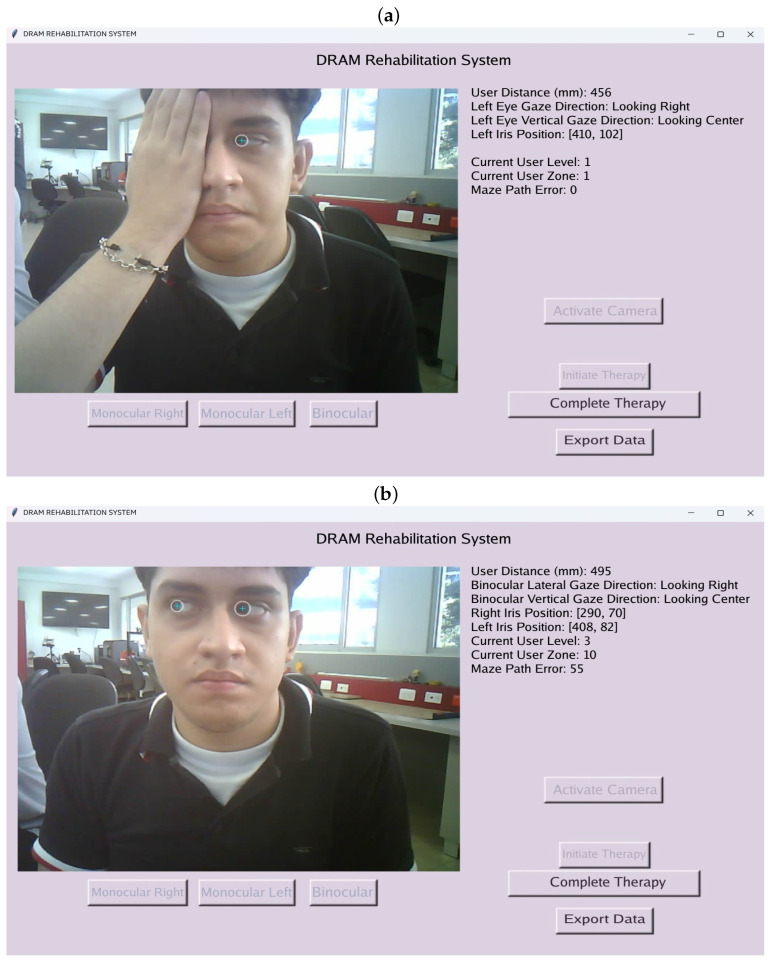
(**a**) Left monocular therapy. (**b**) Binocular therapy.

The interface enables users to select therapy types, such as “Monocular Right,” “Monocular Left,” or “Binocular,” as illustrated in Figure 6. During therapy sessions, companions or specialists can monitor users’ eye movements and associated parameters in real time. These include the user’s distance from the device, real-time pupil coordinates, and gaze direction in both lateral and anteroposterior axes. The figure includes a user who granted approval for their appearance in the publication. In addition, validation tests of the device’s eye-tracking performance are presented later.

To access recorded data, users can navigate to the “Export Data” window, where session-specific and weekly reports are available. Weekly reports include ellipse graphs, tabulated information, and a linear regression analysis illustrating time and error variations.

#### 4.1.1. Functional Test Results

Table 2 summarizes the maximum and minimum values for lateral and anteroposterior displacements (in pixels) across participants, along with the type of therapy conducted, categorized as Binocular (Bin), Monocular Left (Mon-Izq), or Monocular Right (Mon-Der). These values are subsequently visualized using ellipse representations, offering a concise depiction of user progress.

#### 4.1.2. Therapy Performance

Table 3 presents performance metrics for participants tested on level 2 mazes, including checkpoint times, total completion times, errors, and performance ratios. These metrics provide insights into user efficiency and error frequency, enabling graphical representation through linear regression analyses.

The collected data demonstrated therapy durations ranging from 6 to 8 min, aligning with the methodology outlined in the Section 3.4. Elliptical visualizations were selected for their clarity and ease of interpretation compared to time-series displacement graphs, benefiting both users and clinical specialists. In addition, Table 4 and Table 5 show the descriptive statistics of the eye movements obtained in the therapies, highlighting the mean and standard deviation of the segments traveled in level two of the maze. Where HD refers to right lateral displacement, HI to left lateral displacement, and V to anteroposterior upward displacement, all performed throughout level two of the maze.

#### 4.1.3. Case Studies

Two participants with amblyopia were analyzed in detail:

##### User 5

**Figure 7:**
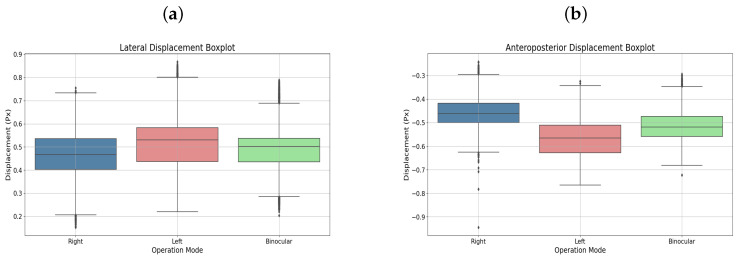
(**a**) Lateral displacement box plot. (**b**) Anteroposterior displacement boxplot. Both corresponding to user 5.

User 5, diagnosed with strabismic amblyopia in the left eye, completed binocular and monocular left-eye therapies. Figure 7a,b display the respective displacements in box plots, highlighting the amblyopic eye’s performance.

**Figure 8:**
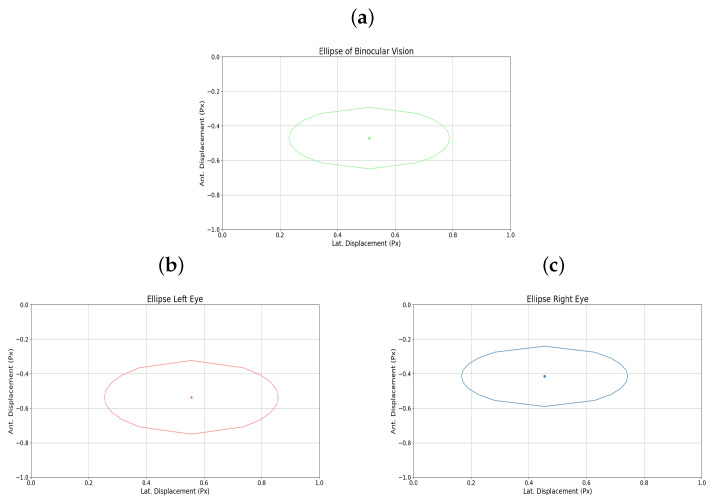
(**a**) Binocular ellipse. (**b**) Ellipse left eye. (**c**) Ellipse right eye. All for user 5.

Additionally, Figure 8a–c shows the elliptical representation of the binocular displacements performed by the user.

**Figure 9:**
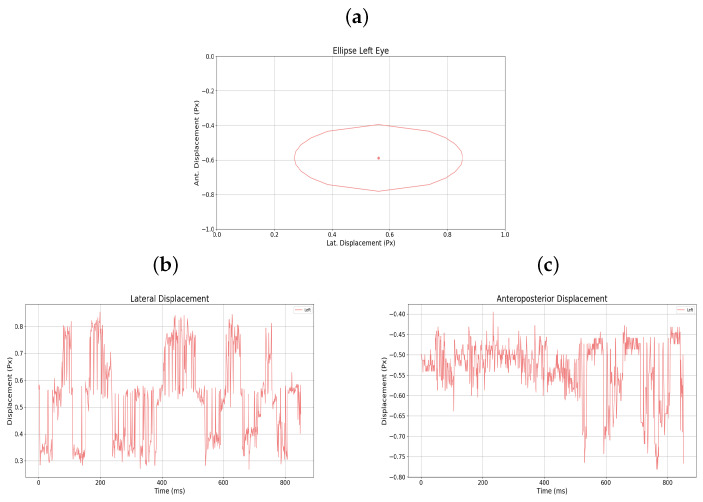
(**a**) Monocular ellipse. (**b**) Lateral displacement. (**c**) Anteroposterior displacement. All for user 5.

The graphical representation of the amblyopic eye is also presented in Figure 9a–c, in this case the left eye, showing the raw displacement data as well as the monocular ellipse generated for that eye.

##### User 15

**Figure 10:**
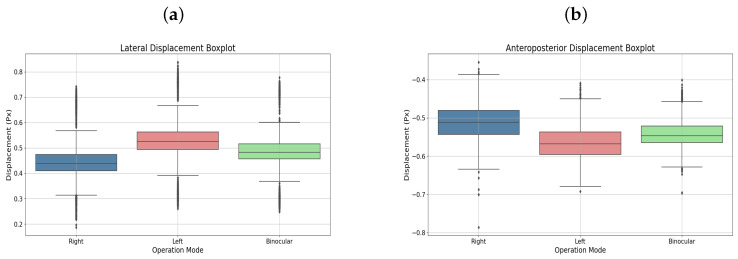
(**a**) Lateral displacement box plot. (**b**) Anteroposterior displacement boxplot. Both corresponding to user 15.

User 15, also diagnosed with strabismic amblyopia, but in the right eye, completed similar therapies. Figure 10a,b present the displacements in box plots, emphasizing progress in the amblyopic eye.

**Figure 11:**
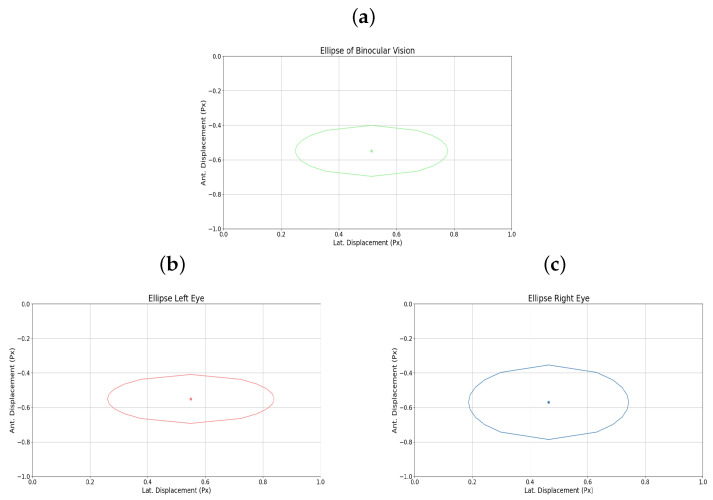
(**a**) Binocular ellipse. (**b**) Ellipse left eye. (**c**) Ellipse right eye. All for user 15.

Additionally, Figure 11a–c shows the elliptical representation of the binocular displacements performed by the user.

**Figure 12:**
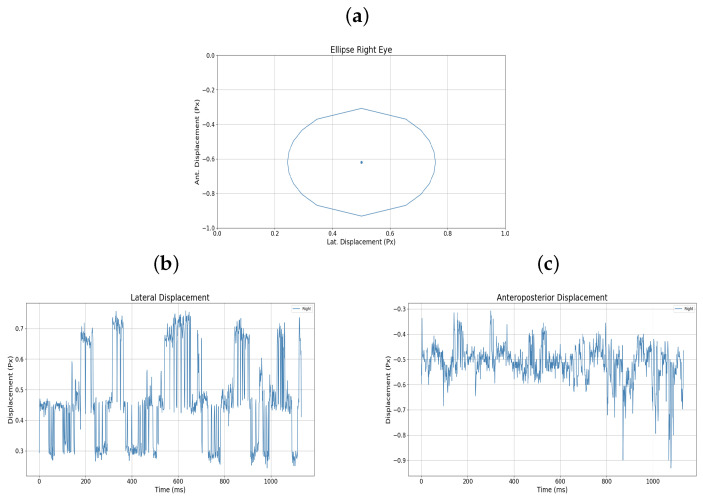
(**a**) Monocular ellipse. (**b**) Lateral displacement. (**c**) Anteroposterior displacement. All for user 15.

The graphical representation of the amblyopic eye is also presented in Figure 12a–c, in this case the right eye, showing the raw displacement data as well as the monocular ellipse generated for that eye.

### 4.2. Device Technical Features

This subsection presents the system’s performance during its implementation, emphasizing its reliability and operational capabilities under real-world conditions. The evaluation considered parameters such as the camera’s viewing angle, the laser sensor’s operating range, the device’s power consumption, and battery performance. These metrics provide insights into the practical functioning of the monitoring system.

The ESP32-CAM microcontroller [55] demonstrated a stable and effective viewing angle of 66 degrees, which facilitated precise environmental monitoring necessary for therapy. Similarly, the VL53L0X laser sensor [52] reliably operated within its specified range of 0 to 1200 mm, ensuring accurate distance measurements crucial for the device’s functionality.

In terms of energy efficiency, the device exhibited a power consumption of 1055 milliwatts when operational. The selected PowerPack, equipped with a 3800 mAh battery, sustained the device for approximately 18 h on a full charge, aligning with the design goal of prolonged usability. This extended operating time underscores the suitability of the chosen power source for continuous therapy sessions without frequent recharging.

A summary of these performance characteristics is provided in Table 6, showcasing the device’s capacity to meet the functional requirements of eye therapy tasks effectively.

To validate the eye-tracking performance of the device, fixation tests were conducted on four healthy users, analyzing the vertices and co-vertices based on the movement of the ocular extremes. It is important to note that the individuals who participated in these tests were the authors of the manuscript. Tests were performed using five fixation points, positioned at the extremes in accordance with the typical gaze targets registered by the device during a standard therapy session.

First, the user fixated on the anteroposterior points, starting from the top, then the center, and finally the bottom. This was followed by fixation on the lateral points in the sequence: right, center, and finally left. At each point, the user was instructed to maintain steady gaze for 10 s.

**Figure 13:**
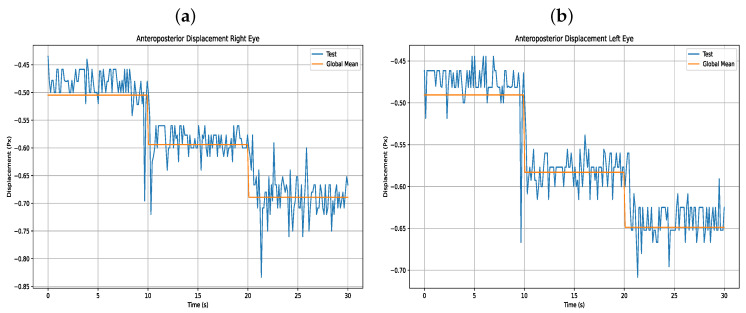
(**a**) Anteroposterior displacement right eye (**b**) Anteroposterior displacement left eye.

Accordingly, five anteroposterior and five lateral trials were conducted for each of the four users. Each movement was segmented and analyzed with its corresponding variable, and statistical calculations were performed, including the mean, standard deviation (STD), and coefficient of variation (CV) for each displacement. The following section presents the results of the users’ anteroposterior displacements, along with corresponding figures from one of the users (User 16), compared against the global average, with statistical details listed in Table 7 and visual data of anteroposterior displacement shown in Figure 13.

**Figure 14:**
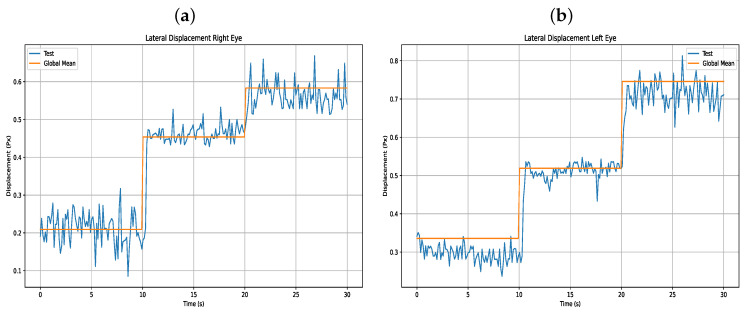
(**a**) Lateral displacement right eye (**b**) Lateral displacement left eye.

Furthermore, Table 8 presents the results of the users’ lateral eye displacements, as shown in Figure 14.

An ANOVA statistical test was also conducted to examine the mean differences in users’ eye movements. Table 9 shows the relationship between each user and their eye movement, including *p*-values and F-values for each variable.

### 4.3. Usability Tests

The usability tests for the visual monitoring device were conducted with a sample selected through non-probability convenience sampling, prioritizing individuals diagnosed with amblyopia or related visual impairments.

#### Perceptions and Opinions

To assess user perceptions of the device, a post-therapy survey was administered using the SUS (System Usability Scale) method. The survey was designed to ensure accessibility, incorporating questions at a reading level suitable for individuals with basic literacy skills (grades 2 and 3 or higher). Additionally, visual aids were included in the Likert scales to enhance comprehension, and the terminology used to reference the device was carefully selected to avoid potential confusion [56].

The results of the survey, detailed in Table 10, capture the usability feedback provided by participants, including three individuals diagnosed with amblyopia.

The survey results indicate the usability scores as well as the visual conditions of each participant. Following the established SUS scoring methodology [57], scores are categorized into three levels: 0–50 as “Unacceptable,” 51–68 as “Marginal” (neutral opinion), and 69–100 as “Acceptable.” Most respondents provided scores in the “Acceptable” range, indicating a generally positive perception of the device’s usability. Participants with visual impairments, including those with amblyopia, reported high levels of satisfaction, suggesting the device met their expectations and requirements effectively. These findings highlight the device’s potential to facilitate visual therapy while maintaining ease of use.

## 5. Discussion

### 5.1. Comparative Analysis with Existing Visual Rehabilitation Approaches

The proposed non-invasive eye movement monitoring tool aligns with current trends in visual rehabilitation that leverage serious games and eye-tracking technologies. For instance, ref. [58] introduced a prototype utilizing infrared sensors to detect pupil movements, aiming to strengthen ocular muscles and provide visual rest for users exposed to prolonged screen time. In their validation study involving 23 participants, 65% reported experiencing eye fatigue, suggesting the prototype’s effectiveness in muscle strengthening. Dichoptic therapies, which employ virtual reality or gaming environments to stimulate the amblyopic eye, have demonstrated improvements in visual acuity (VA). Ref. [59] highlighted the significance of simultaneous and separate stimulation of both eyes to eliminate interocular suppression—a critical factor in amblyopia development. Ref. [29] reported that adults using Oculus Rift VR goggles experienced an average VA improvement of 0.58, with 47% achieving a better corrected VA (BCVA) of 20/40. Occlusive therapies have also shown VA enhancements in both pediatric and adult populations. One study observed an age-related trend in VA improvement, while another found that 68% of patients (13 out of 19) developed binocularity after treatment with Bangerter filters. The NEIVATECH system represents a specific advancement in treating childhood amblyopia by integrating dichoptic training, perceptual learning exercises, and VR components. This method involves distinguishing the orientation of Gabor patches in various VR scenarios, requiring patients to recognize the exact patch presented at the game’s onset [23]. Such approaches open avenues for devices like ours to assist in ocular muscle strengthening, addressing conditions like amblyopia and strabismus.

### 5.2. System Performance and User Interaction

During testing, the implemented pupil tracking system functioned correctly, with no issues in pupil recognition. However, discrepancies arose concerning the thresholds set for controlling the ball within the maze, particularly in estimating vertical gaze. These inaccuracies were often attributed to involuntary head movements during therapy sessions. To mitigate this, users were instructed to maintain an upright posture, positioning themselves 40–50 cm from the device to ensure optimal performance. Therapy sessions conducted on users with amblyopia included both binocular and monocular (right or left eye only) modalities. Data presented in Table 2 illustrate variations in lateral and anteroposterior displacements based on therapy type. For example, User 5, with strabismic amblyopia in the left eye, exhibited greater displacements during left monocular therapy compared to binocular sessions, indicating increased effort by the amblyopic eye. Similarly, User 15 reported heightened fatigue during right monocular therapy, corroborated by displacement records showing increased lateral movement. Users undergoing only binocular therapy did not report eye fatigue impeding their ability to complete the five maze challenges. While some initially struggled to adapt, most quickly acclimated to the device’s operation. Performance metrics, including time to complete mazes and error rates, were recorded. Table 3 details these metrics for level 2 across all participants. Notably, User 5 experienced an increase in collisions during left monocular therapy (43 collisions) compared to the binocular session (35 collisions), despite a reduction in completion time. Consequently, their performance ratio decreased from 4218.62 to 1921.76. User 15, with more advanced amblyopia, showed a significant rise in collisions during right monocular therapy (36 collisions) versus the binocular session (2 collisions), leading to a performance ratio drop from 46,702.41 to 2102.26. Elliptical plots of eye movements further illustrate these findings. For User 5, the left eye’s ellipse during monocular therapy was more pronounced than in the binocular session, reflecting increased effort. User 15’s right eye ellipse showed a marked increase in size during monocular therapy, aligning with their reported fatigue and the advanced stage of their amblyopia.

### 5.3. Technical Evaluation and Variability Analysis

Technical assessments of the device involved calculating mean, standard deviation (STD), and coefficient of variation (CV) for lateral and anteroposterior displacements among users 16 to 19. User 16 displayed a 16.88% variability (0.4154 ± 0.0510) in right eye lateral displacement and 9.64% (0.5336 ± 0.0465) in the left eye, indicating higher variability compared to Users 17 and 19. This increased variability may result from the broader range required for lateral movements. Similarly, User 18 exhibited a 16.46% variability (0.4429 ± 0.0579) in left eye lateral displacement and 10.42% (−0.4476 ± 0.0466) in anteroposterior movement. In contrast, Users 17 and 19 demonstrated lower STDs (approximately 0.15 to 0.2) in lateral movements, suggesting more consistent performance. An ANOVA test revealed high inter-individual consistency across most variables, with *p*-values for the person factor ranging from 0.058 to 0.844. This indicates that ocular movement measurements were similar among users. High *p*-values for movement factors (0.634 to 0.871) suggest that lateral and anteroposterior movements did not significantly affect recorded ratios. Interaction *p*-values ranging from 0.884 to 0.991 further support the consistency of user responses to movement variations. Notably, the variable ratio_V_right exhibited a trend toward statistical significance (*p* = 0.058), which may be attributable to inter-individual differences in ocular morphology or dominance. These results suggest that the device delivers robust and consistent performance across users without the need for individualized calibration, owing to its adaptive thresholding mechanism based on reference points, as illustrated in Figure 1b.

In terms of performance, eye movement validation was carried out using the binocular operation mode, since this requires greater computational processing from the host device for eye monitoring. Additionally, validation was performed in this way because the same algorithm is used for both monocular and binocular tracking. According to the validation Table 7 and Table 8 and the mean plus standard deviation Table 4 and Table 5, the device shows accurate lateral and anteroposterior eye movement. However, in the lateral displacement, there is a tendency for users to deviate the eye with a greater anteroposterior displacement, which may occur due to ocular geometry.

### 5.4. Usability and User Feedback

Usability surveys indicated a high acceptance rate of 86.66% among participants, with the remaining 13.34% expressing a “Marginal” opinion. No participants deemed the device “Unacceptable.” The average usability score was 75.6, with a standard deviation of 9.09, reflecting moderate variability in responses. Users with amblyopia provided acceptable ratings, with User 5 scoring 72.5 and User 15 scoring 77.5. Participants with other visual conditions, such as myopia and astigmatism, reported similar scores—User 3 scored 75, and User 7 scored 77.5 indicating broad acceptability across different visual impairments. Most participants found the device easy to use and intuitive for completing the maze tasks, fulfilling the objective of creating an attractive and user-friendly tool. While some users experienced varying degrees of eye strain, it did not hinder their ability to complete therapy sessions. Notably, eye strain levels correlated with the presence and severity of visual conditions, particularly in amblyopic users.

### 5.5. Addressing Head Movement and Future Enhancements

In this work we only strictly considered eye movement, and a person’s gaze is product of their head position and eye direction, so that a person can look in the same direction by moving only their eyes or moving their head and without moving their eyes. People usually leave their head in a comfortable position before eye orientation, and as a result of this, there arises an investigative problem mentioned in other studies such as ensuring that invariance due to the movement of the head is accounted for. In many of the applications, people tend to move their head or generate fatigue if their head is not in a comfortable position. For these considerations, it is important to take both movements into account, but there are not many studies that consider both movements in a very broad way, despite their importance for clinical diagnoses in ocular diseases [60]. The current prototype assumes minimal head movement during short-duration tasks. However, recognizing that children may introduce significant movement artifacts, future iterations should integrate head tracking technologies, such as infrared or inertial measurement unit (IMU)-based sensors, to compensate for head displacement and enhance gaze estimation accuracy during training sessions. While the current evidence is exploratory and lacks the statistical power to establish therapeutic efficacy, descriptive metrics across users complement individual-case analyses, offering a broader perspective on system functionality and user engagement trends. Importantly, this study does not aim to validate clinical outcomes but to demonstrate the feasibility of a low-cost, gaze-controlled rehabilitation tool for future clinical trials. Future research should focus on controlled efficacy trials to establish therapeutic benefits. Additionally, while the current version prioritizes general usability, future adaptations should aim to make the system more engaging and user-friendly for young children. This includes simplifying the interface and incorporating child-centered visual designs to enhance user experience and compliance.

## 6. Limitations and Future Work

Although the system demonstrates promising usability and technical feasibility, several limitations were identified, and future research directions are proposed below.

### 6.1. Participant Sample and Clinical Validation

One of the primary limitations of the present study concerns the participant sample. Approximately 65% of the participants did not present any diagnosed visual condition. Due to time constraints and limited resources, it was not possible to recruit individuals from the target population, particularly children with amblyopia. Moreover, a non-probabilistic sampling method was used, which restricts the generalizability of the results. Subsequent studies should involve participants with clinically diagnosed amblyopia, especially children within the therapeutic age range. Including both clinical and control groups will be essential to assess the tool’s therapeutic efficacy and clinical relevance.

### 6.2. Usability, Ergonomics, and Pediatric Applicability

Initial testing revealed usability challenges such as involuntary head movements due to lack of experience with the device, and neck strain resulting from extended fixed posture. These factors contributed to user discomfort and tracking inaccuracies. Ergonomic solutions such as head or neck supports will be considered to improve comfort during prolonged sessions. Furthermore, while the System Usability Scale (SUS) was used for adult participants, we acknowledge that these scores may not translate accurately to pediatric populations. Future usability studies will involve child-specific evaluation tools to better assess system practicality for young users.

### 6.3. Visual Interface Design and Accessibility

Although the system interface proved functional, it was not specifically optimized for young children. No formal control was implemented for variations in color vision or the use of corrective lenses, although color transitions in the interface are minimal and non-functional. Future iterations of the system should integrate age-appropriate and engaging visual stimuli to better sustain pediatric users’ attention and enhance adherence. Design improvements may include simplified maze navigation, gamified components, and personalized interaction features based on user age and cognitive profile. Additionally, implementing a larger OLED display is anticipated to facilitate maze visibility for users with advanced visual impairments.

### 6.4. Binocular Function and Stimuli Limitations

The current system does not implement dichoptic stimuli or contrast-balancing features to stimulate binocular collaboration or verify suppression zones. Future enhancements will include contrast-sensitive tasks, dissociative optics, or dual-screen stimuli to better support binocular vision training, which is fundamental in amblyopia rehabilitation.

### 6.5. System Performance and Fatigue Assessment

While the system utilizes MediaPipe and OpenCV for eye-gaze estimation, no formal validation protocol has yet been implemented to benchmark tracking accuracy or error rates. Objective validation of the gaze estimation algorithm will be pursued in future work. Additionally, no structured assessment of fatigue or dropout behavior was conducted during this study. Longitudinal studies will incorporate subjective fatigue questionnaires, dropout metrics, and time-to-task failure tracking to quantify cognitive and physical load, which are critical for long-term therapeutic adherence.

### 6.6. Broader Applications and Societal Impact

Beyond its immediate technical scope, the proposed system may offer significant value in broader clinical and social contexts. Its non-invasive, low-cost, and portable nature positions it as a viable option for use in under-resourced settings. With further refinement, the tool may be integrated into school-based screening programs or remote telemedicine platforms. This would allow for early detection of visual disorders and improved access to rehabilitation, particularly in underserved or rural communities.

### 6.7. Future Preliminary Clinical Validation of the Device

Tests are planned for the future clinical validation of the device over a period of at least one year. This will involve a population of at least 20 children aged between 5 and 10 years old who are able to understand simple instructions and cooperate during visual therapy sessions. They must be diagnosed with unilateral or bilateral amblyopia associated with antisymmetry or strabismus. Before beginning the clinical testing period for the device, an eye exam will be performed by an eye care professional, who will determine the intensity, duration, and mode of operation (monocular therapy of the amblyopic eye, binocular therapy) that the child will use during the therapy session, based on the progression of the condition. These therapy sessions are planned to take place in a neutral space (ideally a medical office) in order to avoid any distractions that could influence the child during the tests. These tests will be conducted under the supervision of a healthcare professional, the child’s legal tutors, and an electronic engineer who will be responsible for verifying the proper functioning of the device. In the future probabilistic sample for the medical validation of the device, subjects with the following characteristics will be excluded, based on [41,61]:

- Children with a history of recent surgery (less than three months prior to the start of the study).
- Presence of eye diseases unrelated to amblyopia, such as pathological nystagmus, congenital glaucoma, color blindness, severe retinopathy of prematurity, or retinal dystrophies.
- Neurological or developmental disorders that interfere with attention, comprehension, or use of the device (e.g., photosensitive epilepsy, severe intellectual disability, ADHD, or autism spectrum disorder at levels 2 or 3).
- Participation in other visual intervention protocols during the last month.
- Voluntary refusal by the child to use the device after the familiarization phase.

## 7. Conclusions

Visual impairment, although not life-threatening, significantly impacts an individual’s quality of life. It restricts social interactions, poses challenges to mobility and transportation, and limits independence and safe ambulation. These factors highlight the need for effective therapeutic solutions, especially those based on technology. However, the involvement of children with electronic devices raises valid concerns that must be addressed. The developed visual monitoring device supports amblyopic eye training by offering a variety of maze challenges, encouraging diverse ocular movements throughout each session. The device records key metrics, such as eye movements, maze completion times, and the distance between the device and the user, which are stored in a database for progress tracking. These data are visualized through ellipse representations, enabling patients to monitor their improvement across sessions. As a non-invasive, session-based therapy, the device aims to help patients gain control over their amblyopic eye. After a week of sessions, patients can assess their performance improvement by reviewing time reductions and errors made at each maze level.

The device is characterized by a long battery life, demonstrated by its low power consumption, as shown in the results. This is a crucial feature, as it allows for multiple therapy sessions to be completed throughout the day without requiring frequent recharging. Additionally, the ESP32-CAM microcontroller camera offers a 66-degree viewing angle, providing an optimal lens for capturing images during therapy sessions. For optimal use, it is recommended that users engage in one therapy session per day, allowing for detailed testing of the device’s functionality.

Another potential improvement is the establishment of a normal range for the ellipses generated during therapy. This would offer reassurance to both patients and specialists regarding the eye’s movement within expected parameters. Further testing is required, both with individuals affected by amblyopia and those without the condition, to refine this metric. Additionally, future versions of the device may include an inertial sensor to compensate for head orientation, which would improve the reliability and accuracy of the collected data. Consultation with specialists will be essential to determine optimal therapy session duration and frequency, as well as to fine-tune the number and design of mazes. Lastly, testing the device with children affected by amblyopia is crucial to validate its effectiveness in real-world conditions and ensure that it meets the needs of its target population.

## Figures and Tables

**Table 1:** Relevant studies on therapy methods for the amblyopia treatment.

Study Title	Implemented Therapy	Results
Outcomes of using Bangerter foils for the treatment of residual amblyopia following patching therapy	Occlusive Therapy This study included a total of 74 patients: 25 patients with strabismic amblyopia, 20 patients with anisometropic amblyopia, and 29 patients with combined amblyopia	The BF treatment led to significant improvements in the visual acuity (VA) of the amblyopic eye without causing reverse amblyopia in any patients. In addition to the improvement in VA, an enhancement in stereoacuity was also observed. Of the 19 patients who initially lacked binocularity, 68% developed it after the treatment. Furthermore, among the patients with poor stereopsis before treatment, 18% showed improvements [27]
Electronically monitored occlusion therapy in amblyopia with eccentric fixation	Occlusive Therapy The study included 12 participants with strabismic and combined amblyopia aged between 2.9 and 12.4 years (mean 6.5). The median occlusion prescription was 7.7 h/day (range 6.6–9.9) and the median daily occlusion received was 5.2 h/day (range 0.7–9.7)	The mean initial acuity of the amblyopic eyes was 1.4 ± 0.4 logMAR (range 0.9–2.0) and that of the contralateral eyes was 0.3 ± 0.3 logMAR (range −0.1–0.8). There was a trend towards better initial amblyopic eye acuity with increasing age, but it did not reach significance (Spearman rank correlation, rho = −0.56, *p* = 0.06). Fellow eyes showed significantly better visual acuity with increasing age [28]
Amblyopia treatment of adults with dichoptic training using the virtual reality Oculus Rift head-mounted display: preliminary results	Dichoptic Therapy A total of 17 subjects (10 males, 7 females) with a mean age of 31.2 years (range, 17–69 years) and anisometropic amblyopia were recruited. Changes in best-corrected visual acuity (BCVA) and stereoacuity were assessed after eight sessions	Mean BCVA in the amblyopic eye improved significantly from a logMAR value of 0.58 ± 0.35 pre-training to a post-training value of 0.43 ± 0.38 (*p* < 0.01). In total, 47% of participants achieved a BCVA of 20/40 or better after training, compared to 30% before training [29]
Rehabilitation of visual functions in adult amblyopic patients with a virtual reality video game: a case series	Dichoptic Therapy A minimum visual acuity difference of 0.2 logMAR (Sloan ETDRS letters) between the two eyes was considered the criterion for amblyopia. Exclusion criteria were the presence of ocular pathologies, such as strabismus and macular or optic nerve disorders, and the use of drugs. The results of one patient will be shown	The participant showed a VA difference of 0.22 logMAR between the contralateral (left) eye and the amblyopic (right) eye at her initial assessment. Initial CS was poor, with only 2 stimuli (out of 8) detected in the low frequency range (3 c/deg). No stimuli were detected at higher frequencies. The stereopsis score showed a value of 160 arc seconds [30]
Serious Games to Support Cognitive Development in Children with Cerebral Visual Impairment	Dichoptic Therapy This study involved children aged between 4 and 9, of whom 13 were girls and 5 were boys. All of these children presented with CVI (Cerebral Visual Impairment), which can lead to the development of amblyopia.	The games developed in this study were very helpful in describing the children’s ability to track moving objects. The system offers various levels of complexity, as it includes different tasks characterized by diverse content. In one of the semantic games, one of the girls achieved 77% correct responses but demonstrated correct performance in her second execution of the task [31]
Rehabilitation of amblyopia using a digital platform for visual training combined with patching in children: a prospective study	Dichoptic Therapy This study included 52 children with amblyopia, of whom 20 improved their VA by combining glasses with patching. In the remaining 32, changes in VA were monitored over a 6-month period.	The study found an improvement in VA of 0.18 ± 0.16 logMAR for the glasses combined with patching group (PG) and an improvement of 0.31 ± 0.35 logMAR for the other visual treatment group (VT). The Wilcoxon effect size was slightly higher in the latter group at 6 months (0.48 vs. 0.54) [32]
P5G: A Patient-Centered Design Method of Virtual Reality Health Game System for Children’s Amblyopia Rehabilitation	Dichoptic Therapy This study presents a P5 eHealth framework applied to health games, through which it develops a VR system for amblyopia rehabilitation in children.	This study does not yet present evaluation results; it proposes two methods for future evaluation: treatment efficacy, where VA will be assessed, and treatment adherence, where usability and playability will be evaluated using the Heuristics Evaluation for Playability (HEP) [33]

**Table 2:** Ocular displacement results (Pixels).

ID	LD Max	AD Max	LI Max	AI Max	LB Max	AB Max	LD Min	AD Min	LI Min	AI Min	LB Min	AB Min	Operation
1	0.71	−0.29	0.90	−0.38	0.81	−0.33	0.03	−0.60	0.29	−0.67	0.20	−0.62	Bin
2	0.70	−0.40	0.79	−0.34	0.75	−0.38	0.12	−0.92	0.29	−0.70	0.21	−0.74	Bin
3	0.76	−0.33	0.85	−0.33	0.76	−0.37	0.18	−0.64	0.24	−0.79	0.24	−0.70	Bin
4	0.73	−0.36	0.81	−0.26	0.79	−0.31	0.18	−0.82	0.24	−0.71	0.23	−0.75	Bin
5	0.74	−0.24	0.86	−0.32	0.79	−0.29	0.17	−0.59	0.25	−0.75	0.23	−0.65	Bin
5	-	-	0.85	−0.40	-	-	-	-	0.27	−0.78	-	-	Mon-Izq
6	0.72	−0.28	0.78	−0.44	0.74	−0.36	0.16	−0.88	0.19	−0.76	0.22	−0.76	Bin
7	0.69	−0.36	0.77	−0.37	0.72	−0.37	0.16	−0.62	0.23	−0.63	0.20	−0.60	Bin
8	0.70	−0.27	0.72	−0.33	0.71	−0.32	0.15	−1.00	0.25	−0.71	0.23	−0.79	Bin
9	0.77	−0.33	0.86	−0.43	0.78	−0.39	0.24	−0.63	0.26	−0.79	0.27	−0.70	Bin
10	0.74	−0.24	0.74	−0.32	0.72	−0.30	0.19	−0.66	0.24	−0.76	0.24	−0.67	Bin
11	0.72	−0.34	0.86	−0.39	0.77	−0.38	0.07	−0.74	0.33	−0.73	0.21	−0.70	Bin
12	0.73	−0.33	0.80	−0.42	0.75	−0.38	0.24	−0.63	0.27	−0.75	0.27	−0.69	Bin
13	0.72	−0.37	0.79	−0.33	0.76	−0.36	0.19	−0.80	0.27	−0.69	0.25	−0.73	Bin
14	0.70	−0.31	0.84	−0.40	0.74	−0.37	0.18	−0.84	0.28	−0.78	0.23	−0.76	Bin
15	0.74	−0.35	0.84	−0.41	0.78	−0.40	0.19	−0.79	0.26	−0.69	0.25	−0.70	Bin
15	0.76	−0.31	-	-	-	-	0.24	−0.93	-	-	-	-	Mon-Der ^1^

^1^ Selected operation mode.

**Table 3:** Therapy session results.

ID	Level	Time-CHKP-1 (ms)	Time-CHKP-2 (ms)	Total Time (ms)	Errors	Performance Ratio (ms/E)
1	2	29,288.28	54,748.03	79,828.34	80	997.84
2	2	24,905.60	52,536.68	94,997.36	34	2794.04
3	2	32,797.24	57,092.38	78,803.16	43	1832.62
4	2	27,144.63	54,854.24	78,771.96	45	1750.49
5	2	25,418.80	122,261.47	147,652.17	35	4218.62
5	2	25,599.37	53,875.00	82,635.54	43	1921.76
6	2	89,477.95	141,704.12	176,117.06	26	6773.72
7	2	30,138.57	55,496.31	81,729.00	34	2403.78
8	2	27,028.62	51,896.15	76,994.55	64	1203.04
9	2	29,031.57	59,622.15	89,168.22	5	17,833.63
10	2	32,637.31	55,022.04	78,409.00	56	1400.16
11	2	30,819.88	59,929.77	87,561.94	33	2653.38
12	2	29,025.76	58,571.12	86,799.60	4	21,699.89
13	2	29,824.20	70,036.41	96,069.72	55	1746.71
14	2	32,495.59	64,573.82	91,083.19	11	8280.29
15	2	28,782.37	65,735.45	93,404.82	2	46,702.41
15	2	26,546.87	50,923.96	75,681.78	36 ^1^	2102.26

^1^ Number of collisions with maze walls.

**Table 4:** Mean ± standard deviation of eye movement in the HD and HI segments.

ID	HD_ML_D	HD_AP_D	HD_ML_I	HD_AP_I	HI_ML_D	HI_AP_D	HI_ML_I	HI_AP_I
2	0.32 ± 0.11	−0.57 ± 0.04	0.42 ± 0.08	−0.48 ± 0.03	0.55 ± 0.05	−0.52 ± 0.03	0.64 ± 0.06	−0.49 ± 0.04
4	0.30 ± 0.08	−0.61 ± 0.38	0.33 ± 0.07	−0.52 ± 0.03	0.62 ± 0.06	−0.54 ± 0.04	0.68 ± 0.09	−0.51 ± 0.05
5 ^1^	0.00 ± 0.00	0.00 ± 0.00	0.36 ± 0.07	−0.50 ± 0.02	0.00 ± 0.00	0.00 ± 0.00	0.70 ± 0.10	−0.51 ± 0.03
5	0.26 ± 0.06	−0.41 ± 0.03	0.33 ± 0.06	−0.48 ± 0.27	0.65 ± 0.08	−0.40 ± 0.19	0.76 ± 0.09	−0.56 ± 0.04
15 ^1^	0.32 ± 0.05	−0.51 ± 0.03	0.00 ± 0.00	0.00 ± 0.00	0.69 ± 0.06	−0.47 ± 0.03	0.00 ± 0.00	0.00 ± 0.00
15	0.32 ± 0.05	−0.51 ± 0.01	0.36 ± 0.07	−0.57 ± 0.01	0.58 ± 0.12	−0.47 ± 0.02	0.67 ± 0.11	−0.60 ± 0.30

^1^ Lateral monocular displacement.

**Table 5:** Mean ± standard deviation of eye movement in segment V.

ID	V_ML_D	V_AP_D	V_ML_I	V_AP_I
2	0.47 ± 0.02	−0.50 ± 0.05	0.54 ± 0.02	−0.45 ± 0.05
4	0.49 ± 0.2	−0.49 ± 0.03	0.53 ± 0.02	−0.45 ± 0.03
5 ^1^	0.00 ± 0.00	0.00 ± 0.00	0.49 ± 0.07	−0.48 ± 0.04
5	0.39 ± 0.10	−0.39 ± 0.03	0.47 ± 0.11	−0.48 ± 0.04
15 ^1^	0.45 ± 0.01	−0.45 ± 0.06	0.00 ± 0.00	0.00 ± 0.00
15	0.44 ± 0.04	−0.45 ± 0.04	0.51 ± 0.04	−0.53 ± 0.05

^1^ Anteroposterior monocular displacement.

**Table 6:** System performance metrics.

Item	Description	Value
ESP32-CAM Camera (Ai-Thinker, CN)	Viewing Angle	66°
VL53L0X Laser Sensor	Operating Range	0–1200 mm
PowerPack (Power supply)	Battery Amperage	3800 mAh
DRAM Device	Power Consumption	1055 mW
DRAM Device	Operating Voltage	5 V
DRAM Device	Approximate Operating Time	18 h ^1^

^1^ Time in hours.

**Table 7:** Validation of anteroposterior displacement.

User	Variable	Mean ± STD	CV (%)
16	ratio_right	0.4223 ± 0.0267	6.37
16	ratio_V_right	−0.5960 ± 0.0340	5.78
16	ratio_left	0.5186 ± 0.0235	4.54
16	ratio_V_left	−0.5741 ± 0.0280	4.95
17	ratio_right	0.4615 ± 0.0211	4.57
17	ratio_V_right	−0.5718 ± 0.0419	7.66
17	ratio_left	0.5200 ± 0.0203	3.92
17	ratio_V_left	−0.5420 ± 0.0394	7.55
18	ratio_right	0.4639 ± 0.0201	4.33
18	ratio_V_right	−0.5027 ± 0.0366	7.39
18	ratio_left	0.4745 ± 0.0245	5.17
18	ratio_V_left	−0.4910 ± 0.0369	7.59
19	ratio_right	0.4696 ± 0.0148	3.16
19	ratio_V_right	−0.5349 ± 0.0244	4.58
19	ratio_left	0.5270 ± 0.0157	3.00
19	ratio_V_left	−0.5451 ± 0.0228	4.18 ^1^

^1^ Percentage value.

**Table 8:** Validation of lateral displacement.

User	Variable	Mean ± STD	CV (%)
16	ratio_right	0.4154 ± 0.0510	16.88
16	ratio_V_right	−0.5934 ± 0.0288	4.85
16	ratio_left	0.5336 ± 0.0465	9.64
16	ratio_V_left	−0.5884 ± 0.0278	4.78
17	ratio_right	0.4570 ± 0.0396	8.85
17	ratio_V_right	−0.5681 ± 0.0405	7.14
17	ratio_left	0.5027 ± 0.0452	9.36
17	ratio_V_left	−0.5482 ± 0.0344	6.30
18	ratio_right	0.4750 ± 0.0469	9.94
18	ratio_V_right	−0.4477 ± 0.0409	9.12
18	ratio_left	0.4429 ± 0.0579	16.46
18	ratio_V_left	−0.4476 ± 0.0466	10.42
19	ratio_right	0.4419 ± 0.0314	7.21
19	ratio_V_right	−0.5390 ± 0.0209	3.91
19	ratio_left	0.5150 ± 0.0281	5.79
19	ratio_V_left	−0.5385 ± 0.0203	3.77 ^1^

^1^ Percentage value.

**Table 9:** ANOVA test.

Variable	F(User)	p(User)	F(Mov)	p(Mov)	F(User-Mov)	p(User-Mov)
ratio_right	0.27	0.844	0.03	0.871	0.04	0.991
ratio_V_right	3.07	0.058	0.24	0.634	0.22	0.884
ratio_left	0.43	0.732	0.06	0.810	0.04	0.988
ratio_V_left	2.77	0.075	0.07	0.796	0.21	0.891 ^1^

^1^ User–movement relationship.

**Table 10:** SUS usability survey results.

Id	Age	Sex	Visual Condition	Score
1	24	Male	No pathology	69 ^1^
2	24	Male	No pathology	75
3	22	Male	Myopia	75
4	22	Male	No pathology	65
5	22	Male	Left Eye Amblyopia	72.5
6	21	Female	No pathology	72.5
7	27	Male	Astigmatism, Myopia	77.5
8	22	Male	Right Eye Amblyopia	90
9	18	Female	No pathology	87.5
10	22	Male	No pathology	70
11	19	Male	No pathology	90
12	25	Female	No pathology	82.5
13	21	Female	No pathology	72.5
14	19	Male	No pathology	57.5
15	24	Male	Right Eye Amblyopia	77.5

^1^ SUS survey score.

## Data Availability

The original datasets presented in this study are included within the article. Any inquiries can be directed to the corresponding authors.

## Abbreviations

The following abbreviations are used in this manuscript:

DRAM	Device for Recording and Analysis in Amblyopia Management
OLED	Organic light-emitting diode
SUS	System usability scale
3D	Three-dimensional
I2C	Inter-integrated circuit
VA	Visual acuity
BCVA	Best corrected visual acuity
WHO	World health organization
